# Evaluation of molecular typing methods for some scab-causing *Streptomyces* strains from Turkey

**DOI:** 10.1007/s11274-024-03914-2

**Published:** 2024-03-05

**Authors:** Kenan Karagoz, Fatih Dadasoglu, Burak Alaylar, Recep Kotan

**Affiliations:** 1 Faculty of Science and Literature, Department of Molecular Biology and Genetics, Agri Ibrahim Cecen University, 04100 Agri, Turkey; 2 Agricultural Faculty, Department of Plant Protection, Ataturk University, 25240 Erzurum, Turkey

**Keywords:** Common-scab, Phylogeny, 16S rRNA, Rep-PCR, ERIC-PCR, BOX-PCR *Streptomyces*

## Abstract

This study was conducted for identifying phylogenetic relationships between 15 scab-causing *Streptomyces* species including *S. bottropensis, S. europaeiscabiei*, *S. scabiei*, *S. stelliscabiei* and, other 11 *Streptomyces* sp. All of the strains were originally isolated from symptomatic potatoes in Erzurum Province, The Eastern Anatolia Region of Turkey. Some morphological and biochemical properties of the strains were defined in our former research. Then, 16 s rRNA regions of them were sequenced. After the sequence data assembly, phylogenetic analyzes were performed. The phylogenetic analyses revealed that the strains are involved in the same major group and, substantially similar to reference strains. Additionally, some subgroup formations were also recorded. Moreover, Repetitive element-based PCR (Rep-PCR), Enterobacterial repetitive intergenic consensus (ERIC-PCR), and BOX-PCR fingerprinting molecular typing methods were used for as molecular typing methods. According to our knowledge, this is the first report on phylogenetic relationships of scab-causing *Streptomyces* species from Turkey. However, the identification of most pathogenic strains remained at the species level.

## Introduction

*Streptomyces* are gram-positive, filamentous bacteria that form extensively branched substrate mycelium. It is also the ability to produce antibiotics or other industrially valuable secondary metabolites (Kumar et al. 2007; Elias et al. 2022) and some of the genus members are used as biocontrol agents against plant disease (Simizu et al. 2009). Some S*treptomyces* species can also cause a plant disease called scab, which is an important problem for potato growers worldwide (Bukhalid and Loria 1997) and the disease significantly reduces the quality of tubers (Gutierrez et al. 2022).

Different phytopathogenic *Streptomyces* species, which are critical hazardous effects on potatoes have been reported all over the world, especially in USA and Canada (Wanner et al. 2009), Sweden (Natsume et al. 2018), China (Liang et al. 2019), Argentina, Mexico, Finland, South Korea, Japan etc. (Shuang et al. 2022).

Scab disease affects some other crops containing beet, carrot, radish, parsnip, and sweet potato (Hill and Lazarovits 2005; Planckaert et al. 2021; Shuang et al. 2022). Moreover, scab-causing *Streptomyces* can damage some seedlings of monocotyledonous and dicotyledonous plants. Injuring of the plant is not necessary for symptoms to occur. Although the symptoms are generally visible on the damaged part of tubers, pathogens can be introduced from lenticels (Wanner 2009). On potato tubers, scab symptoms are variable. Superficial or raised brown spots and dark pits on the skin extending several millimeters into the potato tuber can be observed. The lesions may be small and discrete, or they may coalesce to cover larger areas of the tuber surface (Wanner 2006). Symptom type depends on plant varieties, infection time, the virulence of the pathogen, and environmental conditions.

Three marker genes, *Nec1*, *TomA* (Natsume et al. 2018), and thatxtomin synthesize (*txtA*, *txtB*) were found to be related to the pathogenicity of *Streptomyces* (Zhao et al. 2022). Most of the studies recommended that *Nec1* and *TomA* genes deal with pathogenicity, but these genes are not key factors of pathogenicity (Wanner 2009; Leiminger et al. 2013). Some studies also suggest that different virulence factors may participate in the pathogenicity of *Streptomyces* (Lapaz et al. 2017).

It is also well known that there are plenty of methods for disease control such as cultural, chemical and biological control or resistant varieties. However, in general, commercially insignificant varieties have resistance and none of them is completely resistant (Zadina et al. 1975; Hosny et al. 2014; Sarwar et al. 2018). Moreover, the resistance of the varieties can be alterably related to strains or species of pathogens and soil properties such as pH and moisture etc. (Haynes et al. 1997). Growers generally do not harvest infected tubers and tubers left in fields serve as inoculums for further vegetation (Pavlista 1996). The infected tuber can be accepted more effective than soil inoculums in transferring pathogens. Further, infected tubers significantly transfer novel scab formations of more virulent strains (Loria 2001).

It is well known that there are difficulties arising from some reasons for the taxonomy of Streptomycetes (Hatano et al. 2003; Kim et al. 2012). Therefore, taxonomy and relationships between scab-causing *Streptomyces* spp. have been studied in different ways. Numerical analyses of phenotypic data, fatty acid analyses, and DNA-DNA hybridization (Bouchek-Mechice et al. 2000) are some of these. 16S rRNA gene analysis is also another method with little doubt. The method has some drawbacks like unconformity with DNA-DNA relatedness or heterogeneity among copies within a genome (Kim et al. 2012). Nevertheless, Phylogeny based on 16S rRNA gene sequences has been considered a powerful and promising tool in prokaryote systematic for elucidating phylogenetic relationships among prokaryotic organisms (Stackebrandt et al. 1997) and has been used for as well-known identification of Streptomycetes (Kreuze et al. 1999). In addition to these methods, PCR-based molecular methods have been the center of attraction of scientists. Especially, PCR-based methods of fingerprinting have beneficial role in the existence of repetitive sequences that are distributed bed throughout the genome of distinct bacterial species. For instance, Rep-PCR has been commonly exploited to assessment of the strain specific motifs provided from PCR amplification repetitive DNA fragments exist in bacterial genomes. As an alternative version of Rep-PCR is the amplification of genomic DNA situated among the ERIC-PCR sequences. These sequences are shared along the extragenic regions of the genomes of numerous bacteria (Tajima et al. 2000). On the other hand, BOX-PCR fingerprinting is useful method for typing of diverse bacterial species and it is thought as advantageous complementary instrument for epidemiological researches of members of various type of genus (Tacão et al. 2005).

Our former research showed existence of different *Streptomyces* species causing common scab symptoms on potato in Turkey. This study was designed to the research the relationships between 15 phytopathogenic *Streptomyces* spp., which belong to distinct morphologic groups via 16S rRNA, Rep-PCR, ERIC-PCR and BOX-PCR.

## Materials and methods

### Bacterial strains

All the strains were isolated from symptomatic potato tubers in Erzurum Province, Turkey. Identification of the strains by classical and molecular methods, and characterization of the pathogenicity island (PAI) was performed in our former research (Karagoz 2013; Karagoz and Kotan 2017). Morphological, biochemical properties, and PAI profiles of strains are presented in Table 2.

### Pathogenicity assays

Two different pathogenicity tests were performed. First, potato tuber, cv. marfona, was peeled and sterilized. Disks (2 cm^2^ X 0.5 cm thick) cut from tubers were situated in Petri dishes. Then, strains grown on oatmeal (OM) agar plates were cut and located upside down on the disks. And then, the pathogenicity test of Conn et al. protocol (1998) was performed on the *Streptomyces* species (Conn et al. 1998). Other pathogenicity tests were performed on radish seeds. Briefly, radish seeds were washed and sterilized with 5% sodium hypochlorite for 2 min. Sterilized seeds were placed on Petri plates including 1% water agar. Then, germinated seeds were dipped in bacterial spore suspension at a concentration of ~ 10^9^ CFU / ml. Inoculated seedlings were transferred to tubes containing 1% water agar. Symptoms were evaluated after two weeks (Schaad et al. 2001). Necrosis formation and abnormal growth like dwarfing or hypertrophy are recorded as positive pathogenicity. Tests were repeated three times.

### Sequencing of 16S rRNA genes

16S rRNA gene was amplified by using primers 16S1F and 16S1R. Primer pairs was given in Table 1. The reaction mixture was used according to the Wanner 2006 method. PCR was performed with an Eppendorf gradient PCR thermocycler using the following conditions: an initial denaturation at 95 °C for 5 min, 40 cycles consisting of 94 °C for 20 sn, annealing at 59 °C for 30 sn, and extension at 72 °C for 2 min. Products were run on 1.5% agarose gel. Finally, sequencing was carried out via the dideoxy-chain termination method (Intergen, C.O, Ankara, TURKEY).

**Table 1:** Primers used in this study

	Primers	Reference
*16S rRNA*	16S1F (5’ CATTCACGGAGAGTTTGATCC 3’) 16S1R (5’ AGAAAGGAGGTGATCCAGCC 5’)	Wanner 2006
ERIC-PCR	ERIC 1R (5'-ATGTAAGCTCCTGGGGAT-3') ERIC 2 (5'- AAGTAAGTGACTGGGGGT GAGC-3')	Versalovic et al. 1994
REP-PCR	REP 1R (5'-IIIICGICGICATCIGGC-3') REP 2 (5'-ICGICTTATCIGGCCTAC-3')	Versalovic et al. 1994
BOX-PCR	BOXA1R (5'-CTACGGCAAGGCGACGCTG ACG-3')	Ogutcu et al. 2009

### ERIC, REP and BOX PCR analyses

All the isolates were also characterized by genomic fingerprinting. For this purpose; ERIC, REP and BOX primer sets were used. PCR reactions were prepared according to the former research with some modifications (Versalovic et al. 1994). The primer sets used are presented in Table 1. Briefly, the reaction mixture including; 5 ul 10X PCR buffer without MgCl_2_, 2,0 mM MgCl_2_, 0,4 mM each dNTP’s, 5U Taq DNA polymerase, 0,5 µM each primer and 50 ng template DNA was made up to 50 µl with PCR grade water. PCR was conducted with thermocycler using the following conditions: initial denaturation at 95 °C for 7 min, 30 cycles consisting of 94 °C for 1 min and annealing at 40, 40 and 55 °C for 1 min, for REP, ERIC and BOX primers, respectively; extension at 72 °C for 8 min; a final extension at 72 °C for 15 min. After the PCR, the tubes were cooled at 4 °C. Then PCR products were separated with 1,5% agarose gel and visualized.

### Phylogenetic analysis

**Fig. 1:**
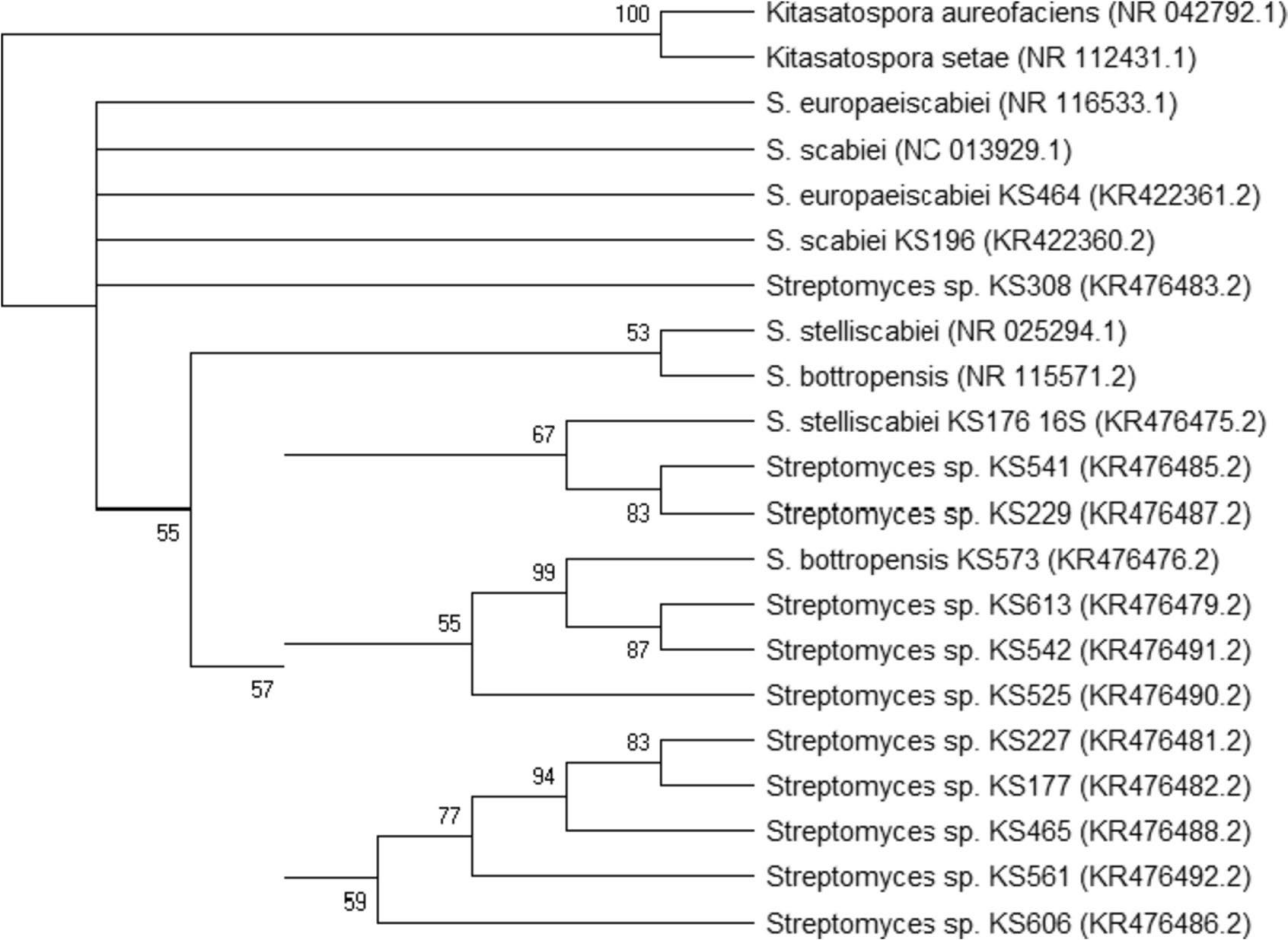
Phylogenetic tree of scab-causing *Streptomyces* spp. based on 16S rRNA regions sequences. (GenBank® accession numbers are presented in brackets)

**Fig. 2:**
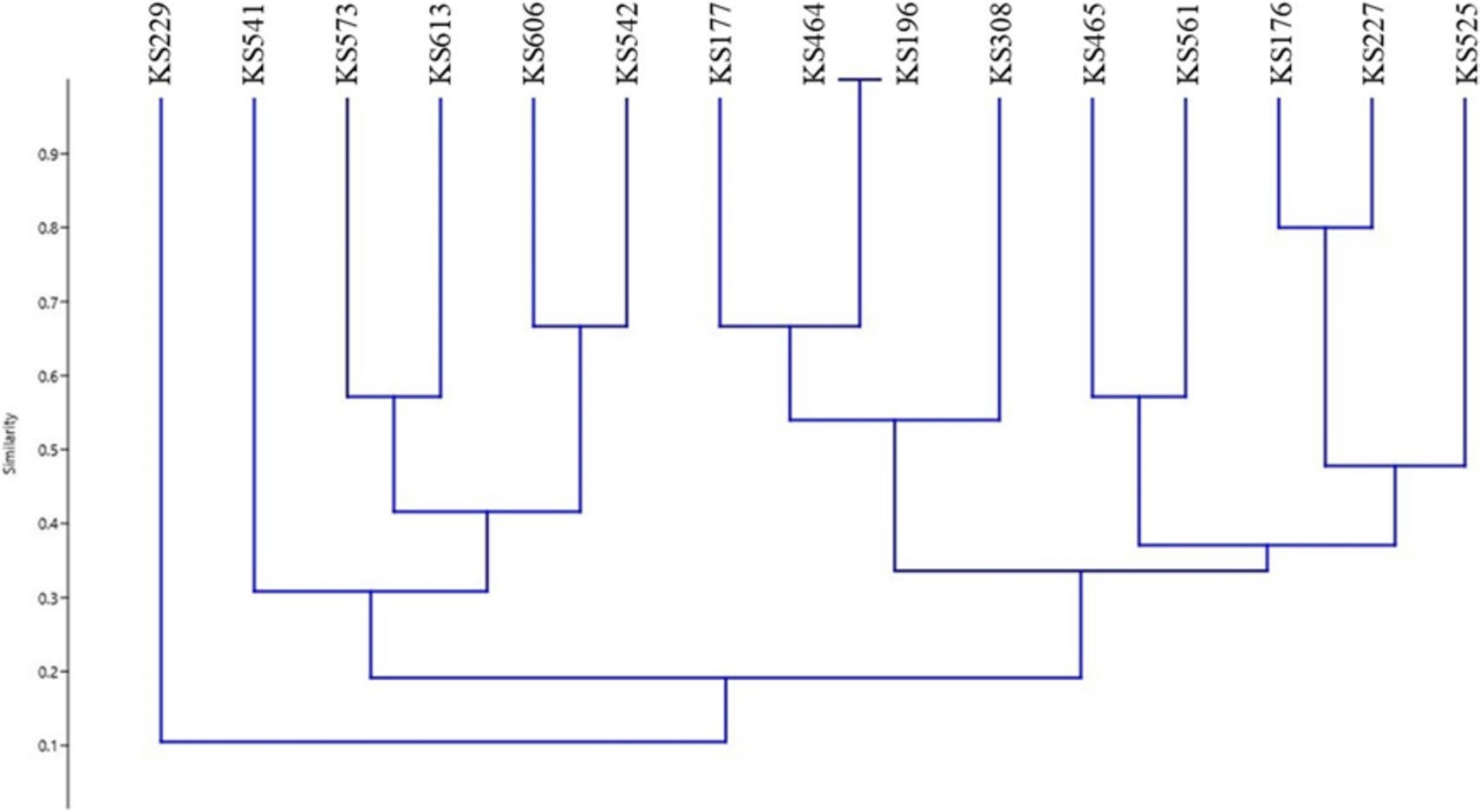
Dendrogram generated from ERIC-PCR banding pattern of 15 *Streptomyces* strains. The similarity analysis was performed with Bray–Curtis and UPGMA method

**Fig. 3:**
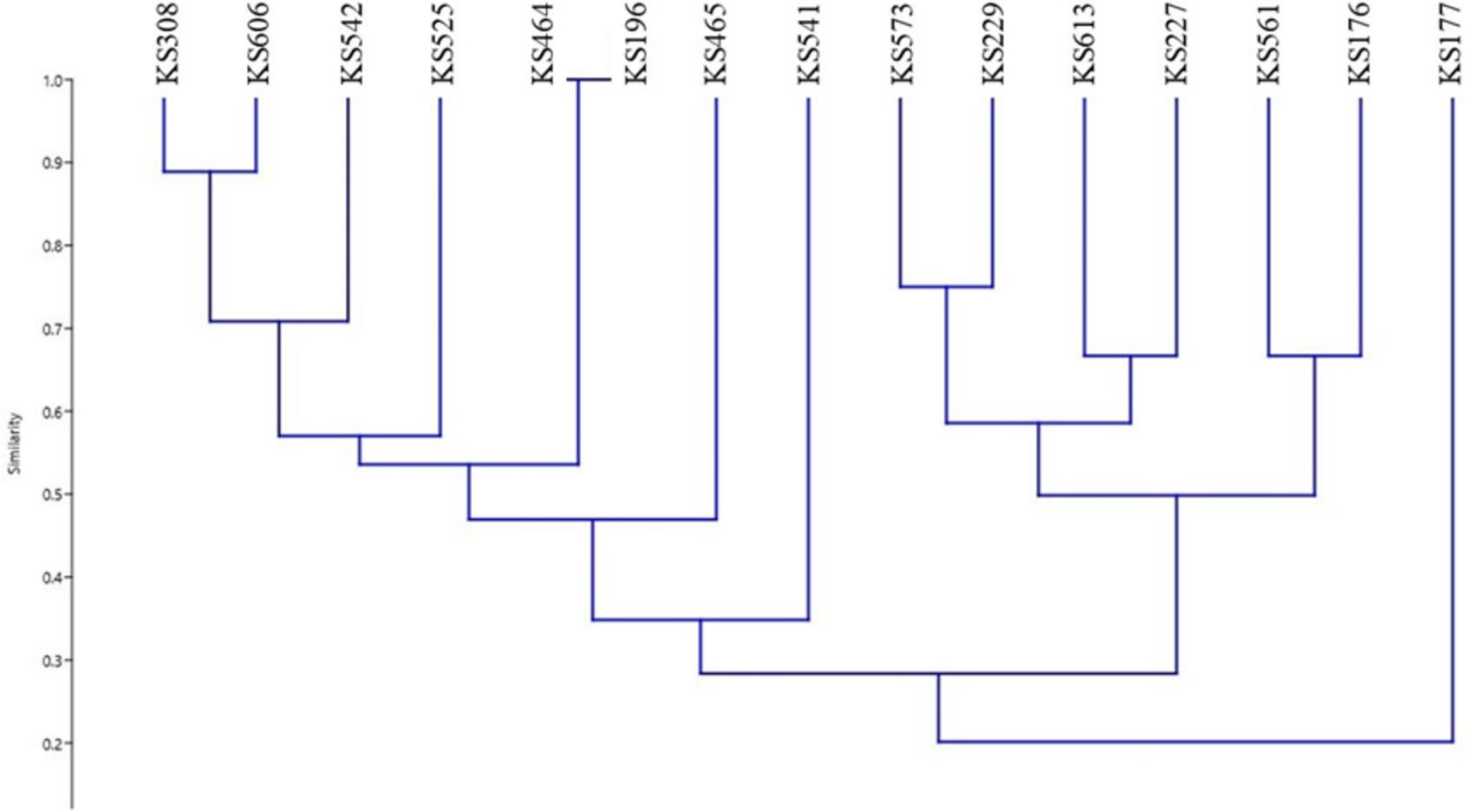
Dendrogram generated from REP-PCR banding pattern of 15 *Streptomyces* strains. The similarity analysis was performed with Bray–Curtis and UPGMA method

**Fig. 4:**
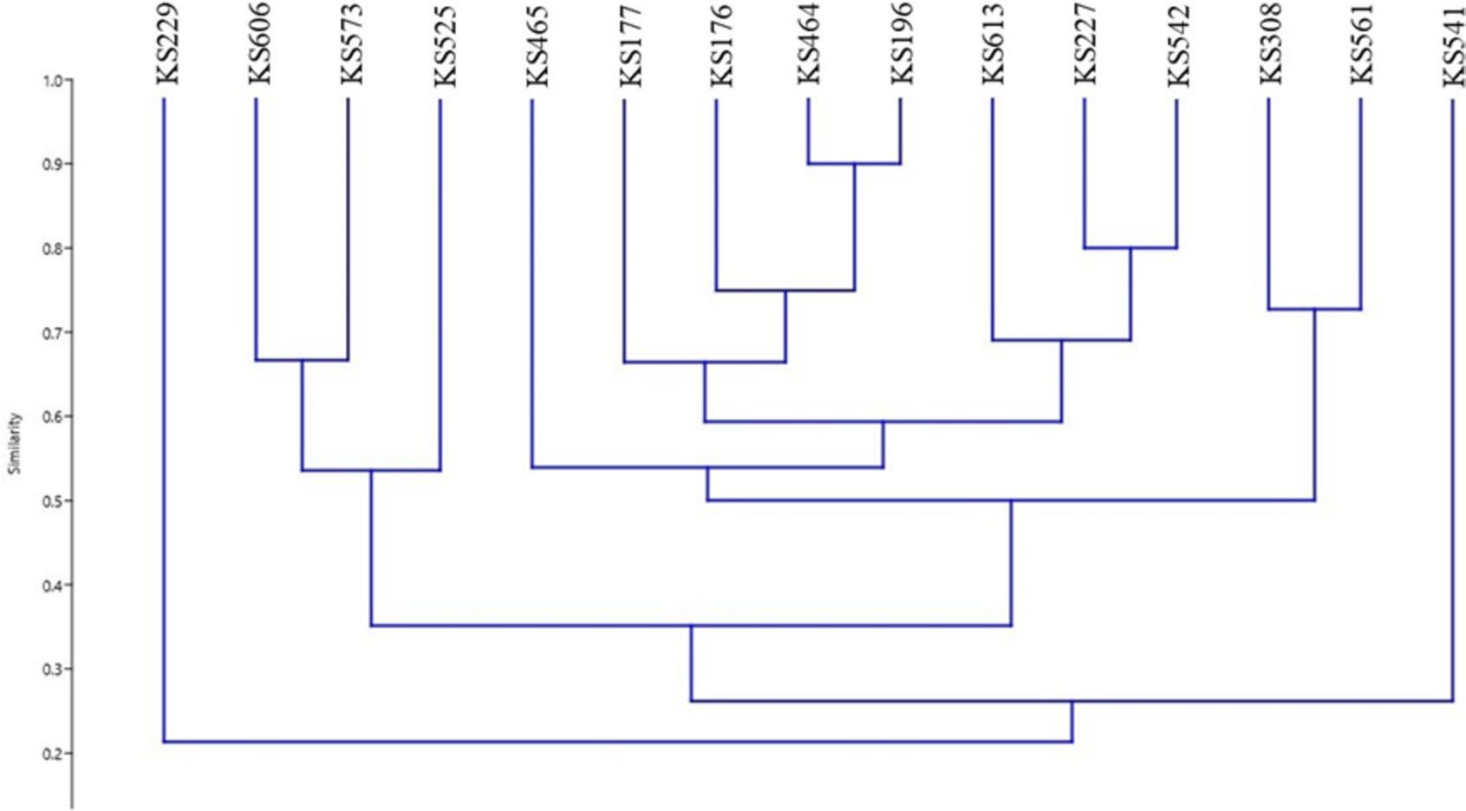
Dendrogram generated from BOX-PCR banding pattern of 15 *Streptomyces* strains. The similarity analysis was performed with Bray–Curtis and UPGMA method

Sequences data were edited and analyzed, using the BioEdit Sequence Alignment Editor 7.0.4.1 software (Hall 1999). All sequence data obtained was confirmed by BLAST searching and was deposited in GenBank® (accession numbers are given in Fig. 1 with brackets). The evolutionary history was inferred using the Neighbor-Joining method. The bootstrap consensus tree deduced from 1000 replicates is provided to represent the evolutionary history of the taxa analyzed. The evolutionary distances were figured out using the Maximum Composite Likelihood technique. All positions including gaps and missing data were removed. There were 1399 positions in the last dataset. Evolutionary analyses were realized in MEGA 6 software (Tamura et al. 2013). 16S rRNA sequence of the reference (*S. bottropensis* NR_115571.2, *S. europaeiscabiei* NR_116533.1, *S. scabiei* NC_013929.1, and *S. stelliscabiei* NR_025294.1) and the out group (*Kitasatospora aureofaciens* NR_042792.1 and *Kitasatospora setae* NR_112431.1) strains were obtained from GenBank®. The banding patterns formed by ERIC- PCR, REP- PCR and BOX-PCR were examined by using Paleontological Statistics Software (PAST). According to the PAST software, the related dendrograms were carried out using an unweighted pair group method with arithmetic mean (UPGMA). Hammer et al. (2001) were used as a reference guideline to analyze for constructed phylogenetic trees of ERIC- PCR, REP- PCR and BOX-PCR data (Fig. 2, 3, 4).

## Results and discussion

When the obtained data were analyzed, all the strains utilized in this study have positive pathogenicity on potato discs and radish seedlings. As well known, pathogenicity test results of tuber slice and radish seedling assays are not parallel at all times (Conn et al. 1998). For this reason, the results were confirmed by both pathogenicity tests. These tests were successfully used in different studies. Although there are exceptions, the results of the pathogenicity tests are generally parallel (Hasani and Taghavi 2014; Lapaz et al. 2017).

Morphological and biochemical test results and marker genes (*Nec1*, *TomA*, and *TxtAB*) in PAI of the strains were determined in our former research (Karagoz 2013). Morphological and biochemical test results (Table 2) are mostly fitted in the literature with a few exceptions. Some variations were observed like resistance to chemicals and antibiotics. Marker genes generally exist in the strains. KS229, KS541, and KS573 strains lack *TxtAB*, KS177 and KS542 strains lack *TomA,* and KS465 strain lacks *Nec1* genes according to PCR results. PAI profiles of strains were presented in Table 2. According to the literature knowledge, it is reported that various pathogenic *Streptomyces* species can be deficient in *Nec1* or *TomA*. Many researchers have mentioned that Nec1 and TomA genes are relevant to pathogenicity but they are not basic determinants of pathogenicity. (Lerat et al. 2009; Park et al. 2003; Leiminger et al. 2013; Dees et al. 2013). Besides, the existence of *Nec1* and *TomA* genes was also reported in non-pathogenic strains (Wanner 2009). Production of thaxtomin was defined as the primary pathogenicity determinant of pathogenic *Streptomyces* species on potatoes in many studies (Wanner 2007a, b, 2009; Flores-Gonzalez et al. 2008; Leiminger et al. 2013). In some studies, however, pathogen *Streptomyces* species, which lack of thaxtomin production ability were also reported (Flores-Gonzalez et al. 2008). Additionally, another study screened that 17% of pathogen strains used in the study did not contain any of the marker genes. Researchers suggest that different virulence factors may participate in pathogenicity (Lapaz et al. 2017) and our findings support this approach.

**Table 2:** Properties and PAI profiles of the strains

Strain number	Identification result	Spore color	Spore morphology	Melanoid pigment production		Streptomycin	Penicillin	Chrystal violet	Phenol	NaCl (%)			Minimum growth of pH	Growth at 37 ºC	PAI profiles		
															*Nec1*	*tomA*	*txtAB*
				PYI	TYR					5	6	7					
KS196	*S. scabiei*	G	S	+	+	–	–	–	–	–	–	–	5,0	+	+	+	+
KS176	*S. stelliscabiei*	G	S	+	+	–	+	–	–	–	–	–	5,5	+	+	+	+
KS464	*S. europaeiscabiei*	G	S	+	+	–	+	–	+	–	–	–	5,0	+	+	+	+
KS573	*S. bottropesnsis*	G	S	+	+	–	+	–	+	+	+	–	4,0	+	+	+	–
KS613	*Streptomyces* sp.	P	R	–	+	–	+	+	+	+	+	+	4,0	+	+	+	+
KS227	*Streptomyces* sp.	G	R	–	+	–	+	–	+	+	+	+	4,0	+	+	+	+
KS177	*Streptomyces* sp.	G	S	–	+	–	+	–	–	+	–	–	5,0	+	+	–	+
KS308	*Streptomyces* sp.	G	S	+	+	–	+	–	–	–	–	–	5,0	+	+	+	+
KS541	*Streptomyces* sp.	G	S	–	+	+	+	+	+	+	+	+	4,0	+	+	+	–
KS606	*Streptomyces* sp.	G	R	–	+	+	+	–	+	+	+	–	4,5	–	+	+	+
KS229	*Streptomyces* sp.	G	S	–	–	–	+	–	+	+	+	+	5,0	+	+	+	–
KS465	*Streptomyces* sp.	G	R	–	+	–	–	–	–	–	–	–	5,0	+	–	+	+
KS525	*Streptomyces* sp.	W	R	+	+	–	+	–	–	–	–	–	4,0	+	+	+	+
KS542	*Streptomyces* sp.	W	R	–	+	–	+	+	+	+	+	+	4,0	+	+	–	+
KS561	*Streptomyces* sp.	G	R	+	+	–	+	+	+	–	–	–	5,0	–	+	+	+

Spore color; G. grey, W: white, P: pale orange. Spore morphology; S: spiral, R. rectiflexous. Phenol: 1%, Chrystal violet: 0,5 µg/ml, Streptomycin 20 µg/ml, Penicillin 10 IU/ml, + : positive, -: negative

For the phylogenetic analysis, 16S rRNA genes, expected ~ 1531 bp size, were cloned by PCR and 1399 bp.16S rRNA gene sequences, between positions in 50 and 1448, were assembled. Coordinated sequence data were analyzed by BLAST. All strains showed 99% similarity with the members of the genus *Streptomyces*. As a result of phylogenetic analyzes, two major groups were obtained. While the out-group strains, *Kitasatospora aureofaciens* and *Kitasatospora setae,* constitute the first group, all the *Streptomyces* species constitute the second group. Formations of some subgroups are also recorded in the second group. *S. scabiei* and *S. europaeiscabiei* were defined as closely related. Positions of *S. scabiei* KS196 and *S. europaeiscabiei* KS464 found to be very close to each other and reference strains (*S. scabiei* NC_013929.1 and *S. europaeiscabiei* NR_116533.1). *S. stelliscabiei* KS176 and *S. bottropensis* KS573 are located in closed positions which are related to reference strains (*S. stelliscabiei* NR_025294.1 and *S. bottropensis* NR_115571.2). Phylogenetic tree of scab-causing *Streptomyces* spp. based on 16S rRNA gene sequences are presented in Fig. 1. Phylogenetic tree derived from 16S rRNA sequence of strains generally show similarity with previous studies. While position of *S. scabiei* and *S. europaeiscabiei* were defined very close, the distance of other strains to them and each other was also recorded as similar (Bouchek-Mechiche et al. 2000; Kim et al. 2012; Park et al. 2003). According to our results, Turkish strains are generally closer to each other.

**Fig. 5:**
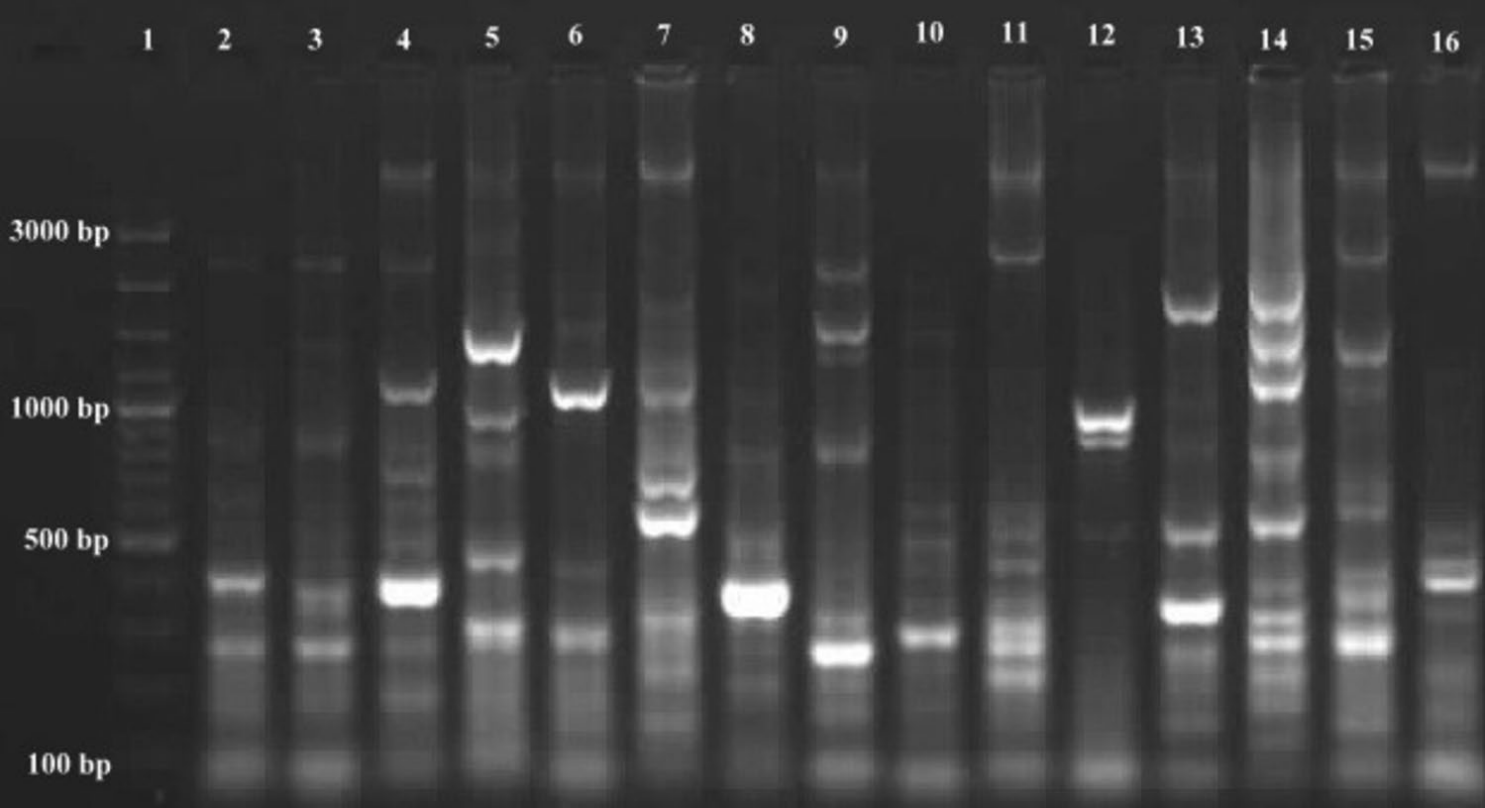
ERIC-PCR band profiles of *Streptomyces* strains with ERIC 1R and ERIC 2 primers. Lanes 1, Marker; 2, KS196; 3, KS464; 4, KS176; 5, KS573; 6, KS613; 7, KS227; 8, KS177; 9, KS308; 10, KS541; 11, KS606; 12, KS229; 13, KS465; 14, KS525; 15, KS542; 16, KS561

**Fig. 6:**
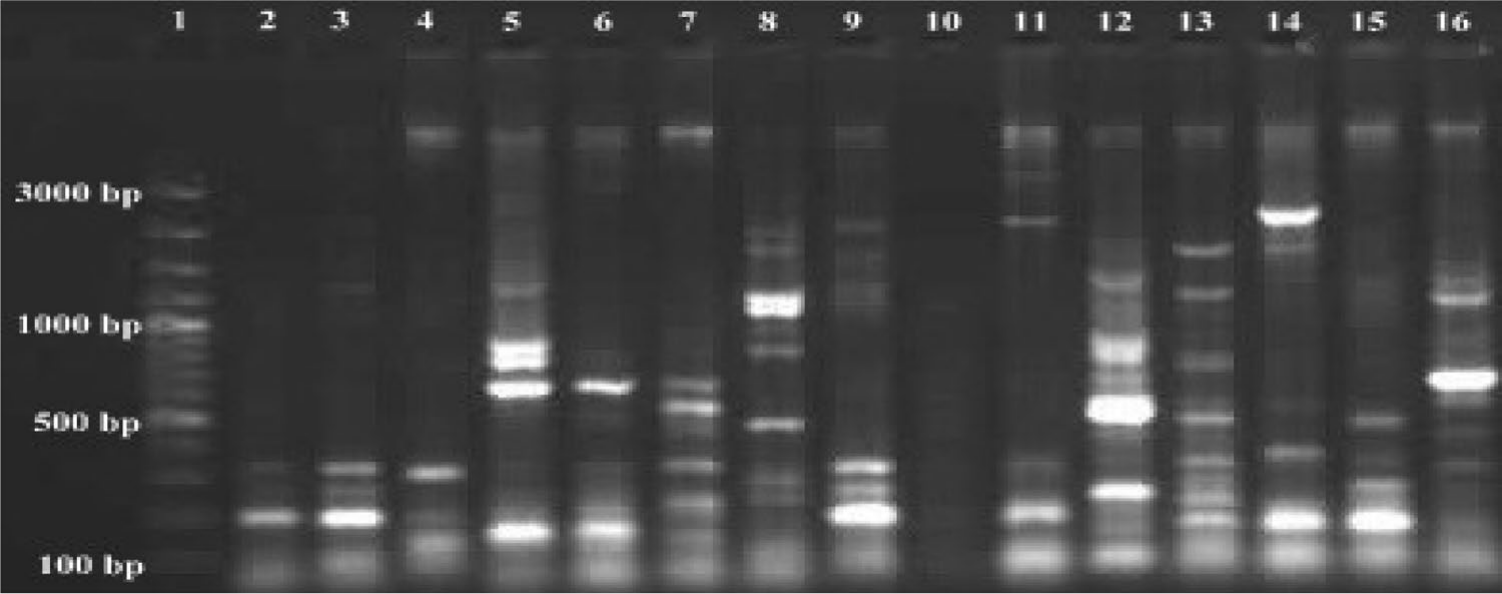
REP-PCR band profiles of *Streptomyces* strains with REP 1R and REP 2 primers. Lanes 1, Marker; 2, KS196; 3, KS464; 4, KS176; 5, KS573; 6, KS613; 7, KS227; 8, KS177; 9, KS308; 10, KS541; 11, KS606; 12, KS229; 13, KS465; 14, KS525; 15, KS542; 16, KS561

**Fig. 7:**
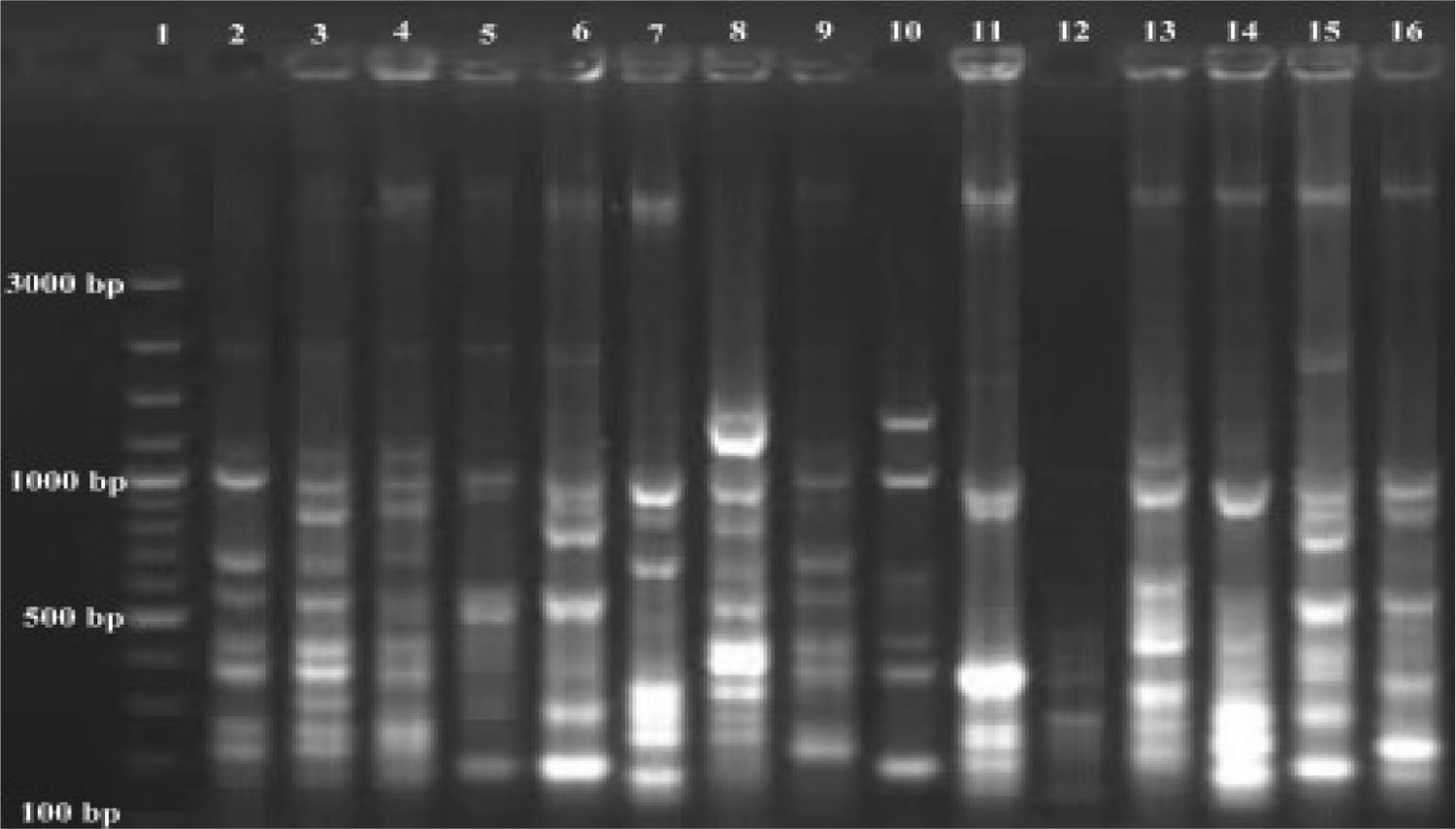
BOX-PCR band profiles of *Streptomyces* strains with BOXA1R primer. Lanes 1, Marker; 2, KS196; 3, KS464; 4, KS176; 5, KS573; 6, KS613; 7, KS227; 8, KS177; 9, KS308; 10, KS541; 11, KS606; 12, KS229; 13, KS465; 14, KS525; 15, KS542; 16, KS561

γ, α and 1435 variable regions were also analyzed. Some variations were observed. Especially γ variable regions have high-value variations in positions 174–202. α Variable region has some variations position in 974–999. It was observed that a few variations in 1435 variable regions position in 1435–1438. Variations in γ, α and 1435 regions of strains are given in Table 3. As a result of the analyzes, the γ region was shown to possess high variability potential than α and 1435 regions. Different formations generally were defined in γ region. *S. scabiei* is also different from *S. europaeiscabiei* in this region. It is known that *S. scabiei* and *S. europaeiscabiei* 16S rRNA regions very similar with just 1 bp mismatch. Mostly similar sequences with former research were detected in γ, α and 1435 regions of strains except for *S. stelliscabiei* KS176 and *S. bottropensis* KS573. Some differences were encountered in γ, α and 1435 regions of *S. stelliscabiei* KS176 and *S. bottropensis* KS573, when compared to literature (Wanner 2006). We think that some changes in 16S rRNA sequence could be possible depending on conditions (locations and climates etc.) because of their genetic variation potential. These strains were identified by classical methods (given in Table 2.) and PCR analyzes were also performed by using specific primer pairs Stel3/T2st2 and Stel3/Aci2 (Wanner 2006) for *S. stelliscabiei* and *S. bottropensis,* respectively in our former studies (Karagoz 2013; Karagoz and Kotan 2017). Specific DNA bands were observed for both *S.stelliscabiei* and *S. bottropensis*. Moreover, the ERIC primer set formed reproducible and distinct fingerprints containing 3–9 fragments between 100 and 3000 bp. REP PCR demonstrated that *Streptomyces* strains have different patterns with 2–8 fragments ranging from to 100 3000 bp. For BOX-PCR fingerprint showed 2–11 fragments in the size of 100-3000 bp (Figs. 5, 6, 7).

**Table 3:** Genetic variations in γ, α and 1435 variable regions of the strains

Strains		γ—variable region Position in 174–202	α—variable region Position in 974–999	1435 variable region Position in 1435–1438
KS196 *S. scabiei*	(KR422360.2)*	CGACACTCTCGGGCATCCGATGAGTGTGG	ACACCGGAAACGGCCAGAGATGGTCG	GTAA
KS464 *S. europaeiscabiei*	(KR422361.2)	CAACACTCTCGGGCATCCGATGAGTGTGG	ACACCGGAAACGGCCAGAGATGGTCG	GTAA
KS176 *S. stelliscabiei*	(KR476475.2)	CTATCGCCTTGGGCATCCTT-GGTGATCG	ACACCGGAAAGCATCAGAGATGGTGC	TTGT
KS573 *S. bottropensis*	(KR476476.2)	ACACTTCTGCTCTCATGGGC-AGGGGTTA	ACACCGGAAAGCATCAGAGATGGTGC	TTGT
KS613 *Streptomyces* sp.	(KR476479.2)	ACACTTCTGCTCTCATGGGC-AGGGGTTA	ACACCGGAAAGCATCAGAGATGGTGC	TTGT
KS227 *Streptomyces* sp.	(KR476481.2)	ACACTCTGTCCCGCATGGGA-CGGGGTTA	ATACCGGAAAGCATCAGAGATGGTGC	TTGT
KS177 *Streptomyces* sp.	(KR476482.2)	ACACTCTGTCCCGCATGGGA-CGGGGTTA	ATACCGGAAAGCATCAGAGATGGTGC	TTGT
KS308 *Streptomyces* sp.	(KR476483.2)	ACACTCTCTCGGGCATGGGATGAGTGTGG	ACACCGGAAACGGCCAGAGATGGTGC	GTAA
KS541 *Streptomyces* sp.	(KR476485.2)	CTACCCGCTTGGGCATCCAA-GCGGTTCG	ACACCGGAAAGCATTAGAGATGGTGC	TTGT
KS606 *Streptomyces* sp.	(KR476486.2)	ATACTTTCCCTCTCATGGGG-GAAGGTTA	CGCCCGGAAAGCCGTAGAGATGGTGC	TTGT
KS229 *Streptomyces* sp.	(KR476487.2)	CTACGCGCTCAGGCATCTGATGCGCGTGG	ACACCGAAAAACTTTGGAGACAAGGC	TTGT
KS465 *Streptomyces* sp.	(KR476488.2)	ACACTCTGTCCCGCATGGGA-CGGGGTTA	ATACCGGAAAGCATCAGAGATGGTGC	TTGT
KS525 *Streptomyces* sp.	(KR476490.2)	ACACTGCCACGGGCATCTGT-GGTGGTTA	CGCCCGGAAAGCATCAGAGATGGTGC	TTGT
KS542 *Streptomyces* sp.	(KR476491.2)	ACACTCCTGCTCTCATGGGC-AGGGGTTA	ACACCGGAAAGCATCAGAGATGGTGC	TTGT
KS561 *Streptomyces* sp.	(KR476492.2)	ACACCGGCTTCCGCATGGGA-GCTGGTTG	ATACCGGAAAGCATTAGAGATGGTGC	TTGT

^*^GenBank® accession numbers are given in brackets

Numerous methods use for determining to the molecular diversity of scab-causing Streptomyces species. ERIC-PCR, REP-PCR and BOX-PCR have unique and promising discriminatory methods and a rapid and relatively simple comparative methods, making them beneficial for procedure epidemiological studies (Bakshi et al. 2018). In this study, ERIC-PCR, REP-PCR and BOX-PCR were also used as discriminatory methods. As it is expected, the analyzes of the ERIC-PCR, Rep-PCR, and BOX-PCR data showed distinct phylogenetic patterns. All the three methods precisely demonstrated that KS196 *S. scabiei* and KS464 *S. europaeiscabiei*, closely related species, positioned and classified mutual group. On the other hand, the other strains were determined in various phylogenetic positions according to the exploited PCR methods. Among these methods, when we compared ERIC-PCR and 16S rRNA PCR results, the phylogenetic patterns have high level of similarity between each other. In both analyses, closely related strains were situated in similar positions. It was observed that Rep-PCR, and BOX-PCR methods were insufficient to locate the strains determined to be related according to ERIC-PCR and 16S rRNA PCR analyses. Considering all the data, it is thought that ERIC-PCR method may be useful in phylogenetic analyzes of Streptomyces species as an auxiliary tool.

Consequently, 15 different scab-causing *Streptomyces* species from Turkey were identified and analyzed based on 16S rRNA sequences. The results in the current study clearly showed that ERIC-PCR, Rep-PCR, and BOX-PCR fingerprinting molecular typing methods are useful and safe methods for the investigation of *Streptomyces* strains isolated from symptomatic potato tubers. According to our knowledge, this is the first report on phylogenetic analysis of scab-causing *Streptomyces* species in Turkey. However, most of the pathogenic strains remain to be identified at the species level.

## Conclusion

According to the literature, there are numerous unknown local pathogenic microorganisms. Hence, it is important to know pathogenic isolates in soil systems to struggle and overcome to these problems for sustainable agricultural productivity. Therefore, when more pathogenic strains are identified for species level, it will be helpful for control of the various pathogenic strains in agroecosystems.

## Data Availability

Sequencing data are openly available in the NCBI database.
